# Pattern of Facial Laceration at Tertiary Care Centre in Eastern Nepal: A Descriptive Cross-sectional Study

**DOI:** 10.31729/jnma.8537

**Published:** 2024-04-30

**Authors:** Gopal Gurung, Mona Pokharel

**Affiliations:** 1 Department of Dental Surgery, Birat Medical College and Teaching Hospital, Tankisinwari, Morang, Nepal

**Keywords:** *face*, *injuries*, *lacerations*

## Abstract

**Introduction:**

Facial lacerations are a source of concern as these can be life threatening at times due to extensive haemorrhage and also leave lifelong scars. The objective of this study was to find out the pattern of facial lacerations in the Nepalese population visiting a tertiary care centre in eastern Nepal.

**Methods:**

A descriptive cross-sectional study was conducted among the maxillofacial trauma patients visiting the Emergency department and department of Dental Surgery at a tertiary care centre from 1 October 2022 to 30 September 2023. Ethical approval was taken from the Institutional Review Committee . All patients attending the Dental outpatient department and Emergency department for the management of facial laceration in the study period were included in the study.

**Results:**

Out of 236 patients, there were 199 (84.32%) male and 37 (15.67%) female patients. The most common age group was of 21-30 years 88 (37.29%) and Road Traffic Accidents 183 (77.54%) was the main aetiology. Facial lacerations and maxillofacial fractures both were seen in 98 (41.53%) patients. There were a total of 358 facial laceration sites among 236 patients and chin region 76 (21.22%) was the most common followed by forehead region 54 (15.08%).

**Conclusions:**

Facial lacerations were mostly seen in males, younger adults and road traffic accidents were the main aetiology for these injuries. Facial lacerations showed predominant T-shaped distribution with chin being the most common site.

## INTRODUCTION

Maxillofacial trauma may present as soft tissue injuries or fractures of facial bones or both. Facial lacerations are a source of concern as these can be life threatening at times due to extensive haemorrhage and also leave lifelong scars. In western countries falls and assaults are main aetiology for facial laceration but in Asia and Africa it is Road Traffic Accidents (RTA).^1,2^ Lacerations are more frequently localised to the middle third of the face and "T-shaped" area including forehead, nose, lips, and the perioral area.^3,4^

Studies in Nepal have shown laceration as the most common soft tissue injury of the face with the prevalence of 59.8% to 70.7%.^5,6^ However, there is a lack of literature on the pattern of Facial laceration in the Nepalese population.

The objective of this study was to find out the pattern of facial lacerations in the Nepalese population visiting a tertiary care centre in eastern Nepal.

## METHODS

This descriptive cross-sectional study was conducted among the maxillofacial trauma patients visiting the Emergency Department and Department of Dental Surgery at Birat Medical College and Teaching Hospital, Tankisinwari, Morang, Nepal from 1 October, 2022 to 30 September, 2023. Ethical approval was obtained from the Institutional Review Committee (Reference number: IRC-PA-229/2078-79). Written informed consent was acquired from the patients or their visitor depending on the patient's condition and age. All patients attending Dental outpatient department and Emergency department of Birat medical college for management of maxillofacial laceration in the study period were included in the study. Patients without any form of identification (unknown) or those not willing to be enrolled in the study were excluded. The sample size was calculated using the formula:


$$\begin{array}{ll} \text{n} & ={\text{Z}}^{2}\times \frac{\text{p}\times \text{q}}{{\text{e}}^{\text{2}}}\\ & =1.96^{2}\times \frac{0.50\times 0.50}{0.07^{2}}\\ & =196 \end{array}$$

Where,

Z = 1.96 at 95% Confidence Interval
p = 0.5 at 50% prevalence assumption of facial laceration among maxillofacial injuries
q = 0.5
e = 0.07 at 7% margin of error

The minimum required sample size was 196. However, the final sample size taken was 236.

Demographic data of the patients were entered in the designed proforma from interview after the examination of facial laceration itself. Etiology of facial laceration, the influence of alcohol, other associated injuries, and site of facial laceration were recorded in paper based form. Blood alcohol concentration test was not done for alcohol influence, it was recorded based on history of patient and odour. Facial lacerations were recorded using the MCFONTZL system developed by Lee et al. with modifications made as required.^7^ Scalp, ear and intraoral lacerations were excluded.

Data were entered in Microsoft Excel and analysed using IBM Statistical Package for the Social Sciences. Frequencies and percentages were used to represent data.

## RESULTS

Among 236 patients, there were 199 (84.32%) male patients and 37 (15.68%) female patients. Age of the patients ranged from 11 months to 84 years with the greatest number of patients seen in the age group of 21-30 years 88 (37.29%) (Table 1).

**Table 1:** Frequency of facial laceration according to age group (n= 236).

Age range (years)	n (%)
0-10	13 (5.51)
11-20	37 (15.68)
21-30	88 (37.29)
31-40	38 (16.10)
41-50	31 (13.14)
51-60	19 (8.05)
>60	10 (4.24)

The most common aetiology was Road Traffic Accidents (RTA) 183 (77.54%) and among them motorcycles 135 (57.20%) accounted for the majority of the cases (Table 2). Others accounted for 2.5% only which contained animal attack and injury at the working site.

**Table 2:** Aetiology of Facial laceration (n = 236).

Aetiology		n (%)
	Four wheeler	10 (4.24)
Road traffic accidents (n=183)	Three wheeler	8 (3.39)
	Motorcycle	135 (57.20)
	Bicycle	11 (4.66)
	Pedestrian	19 (8.05)
Assault		22 (9.32)
Fall		25 (10.59)
Others		6 (2.54)

Alcohol influence was associated with 93 (39.41%) patients (Table 3). Other associated body injuries were seen in 73 patients and among them most common was head 35 (47.94%) associated injury. Facial lacerations and maxillofacial fractures both were seen in 98 (41.53%) patients.

**Table 3:** Frequency of facial laceration according to alcohol influence, associated injuries and maxillofacial fracture (n= 236)

Variables		n (%)
Alcohol influence	Yes	93 (39.41)
	No	143 (60.59)
Associated injuries (n=73)	Head	35 (47.94)
	Orthopaedics	34 (46.58)
	Chest	2 (2.74)
Abdomen	2 (2.74)
Maxillofacial fracture	Yes	98 (41.53)
	No	138 (58.47)

There were a total of 358 facial laceration sites among 236 patients. Among facial lacerations, the lacerations in chin region was 76 (21.22%) followed by forehead region 54 (15.08%), maxilla 40 (11.17%), right zygoma 36 (10.06%), right orbit 27 (7.54%), upper lip 26 (7.26%), left orbit 25 (6.98%), nose 21 (5.87%), left zygoma 20 (5.59%), lower lip 19 (5.31%), right temporal 8 (2.23%) and left temporal 6 (1.68%) respectively.

## DISCUSSION

In this study, male patients that presented with facial laceration were 199 (84.32%) while female accounted for 37 (15.68%) with a ratio of 5.3:1 which is higher as seen in other studies.^1,8,9^ This may be due to our society being patriarchal, males have more responsibilities and have to go outdoor more, have an active social life with alcohol consumption that exposes them more to maxillofacial injuries.^6,10^

The maximum number of patients was seen in the age group of 21-30 years 88 (37.29%) which is similar to other studies.^9,11^ This age group is active and have more responsibilities for the livelihood of their families and also have phase of great personal independence, social excitement, and exposure to violence as well.^12,13^

The most common aetiology was RTA 183 (77.54%) followed by falls 25 (10.59%) which is in contrast to study by Bolt et al. where most common causes of facial laceration were falls 56.3% and assaults 15.5%.^1^ RTAs was only 3.7% which shows the difference in the aetiology of developed and and developing countries. Study by Ong et al. in the United kingdom shows falls 44% and assaults 35% as the major aetiology for soft tissue injuries of the face with RTA being only 5%.^14^ According to Hussaini et al. in Malaysia, RTA 75% and falls 16% were the common aetiology of soft tissue injuries of the face which is similar to our study findings.^9^ Among RTA, motorcycle 135 (57.20%) was the most common vehicle involved which is similar to Hussaini et al 40%.^9^

Alcohol influence was identified in 93 (39.41%) patients which is more than that seen in study by Pradhan et al. 10%.^5^ This may be due to change in the drinking habits of the population as drinking is much more socially acceptable now compared to 10 years back when the study by Pradhan et al. was done. Alcohol's has a depressant effect on the central nervous system that reduces the cognitive ability to assess risk, and reduces the ability to make rational decisions.^15^ There have been associations between alcohol consumption and maxillofacial injuries.^12^

Head injuries were the most common 35 (47.94%) associated with maxillofacial lacerations, consistent to the other studies.^5,6^ Both Facial laceration and maxillofacial bone fracture were seen in 98 (41.53%) patients similar to study by Rocia et al 32.5%.^3^

Out of 236 patients, facial laceration was seen in 358 sites. Facial laceration showed predominant T-shaped distribution, over the chin 76 (21.22%), forehead 53 (15.08%), orbit (52 (14.52%), maxilla 40 (11.17%) and upper lip 26 (7.26%) similar to other studies.^1,3,4,8^ Within T-shaped distribution also there was difference, chin region 76 (21.22%) was the most common site in our study compared to studies by Bolt et al, Mo young et al. and Lee et al. where the forehead region was the most common site. This could be due to the change in the aetiology of the maxillofacial laceration as RTA was main aetiology in our study however in above mentioned studies main aetiology was fall. As stated by Bolt aetiology has a profound influence on the distribution of facial lacerations.^1^ Chin being the most common site is similar to the study by Roccia et al. when RTA and fall both were taken into the count as aetiology.^3^ T-shaped distribution is directly associated with the anatomic bony prominences and in response to blunt trauma, skin breaks along selected lines of least resistance that closely parallel cleavage lines of the face.^16^ Lee et al. suggested that the skin is more likely to lacerate when the underlying bone can resist the forces that could produce a fracture. Compared to other facial bones mandible and frontal bone better resist fracture from the blunt trauma leading to the more force distribution on the overlying skin, increasing the likelihood of laceration.^16^

## CONCLUSIONS

Facial lacerations were mostly seen in males, younger adults and road traffic accidents were the main aetiology for these injuries. Facial lacerations showed predominant T-shaped distribution with chin being the most common site.
